# The Influence of Treatment With Sofosbuvir/Velpatasvir on Children’s Growth—Results of the PANDAA-PED Study

**DOI:** 10.1097/INF.0000000000004504

**Published:** 2024-09-04

**Authors:** Maria Pokorska-Śpiewak, Ewa Talarek, Małgorzata Aniszewska, Magdalena Pluta, Anna Dobrzeniecka, Magdalena Marczyńska, Giuseppe Indolfi

**Affiliations:** * From the Department of Children’s Infectious Diseases, Medical University of Warsaw, Warsaw, Poland; † Department of Pediatric Infectious Diseases, Regional Hospital of Infectious Diseases in Warsaw, Warsaw, Poland; ‡ Department of Neurofarba, Meyer Children’s University of Florence, Florence, Italy; § Meyer Children’s Hospital, Istituto di Ricerca e Cura a Carattere Scientifico, Florence, Italy.

**Keywords:** body mass index, children, growth, hepatitis C, sofosbuvir/velpatasvir

## Abstract

**Background:**

The aim of this study was to evaluate the influence of treatment of hepatitis C with sofosbuvir and velpatasvir (SOF/VEL) on children’s growth.

**Methods:**

Fifty children 6-18 years of age were successfully treated for hepatitis C with a 12-week course of SOF/VEL fixed dose adjusted to the body weight in the PANDAA-PED (Treatment of chronic hepatitis C in children 6–18 years of age using a pangenotypic direct-acting antiviral sofosbuvir/velpatasvir) project. Growth parameters were compared at 1 year after treatment with baseline (at the start of treatment) and 12-week-posttreatment values. Body mass index (BMI), weight and height Z scores adjusted to sex and age were calculated according to the World Health Organization reference data.

**Results:**

Forty-nine participants (23 boys and 26 girls) completed all the visits. The mean age at 1 year after treatment was 10.9 ± 2.5 years, and all children had undetectable hepatitis C virus RNA at this point. Significant weight and height gains were observed after treatment irrespective of the patients’ age and sex. Height Z scores did not vary significantly both at 12 weeks and 1 year after treatment, confirming a normal increase in participants’ height. Weight Z scores for 16 children below 10 years of age decreased at 1 year after treatment. BMI Z score values decreased at 12 weeks after treatment compared to the baseline in boys, but no difference was found between 1-year posttreatment and baseline BMI Z scores in both girls and boys.

**Conclusions:**

Results of the PANDAA-PED study showed normal growth up to 1 year after successful treatment with SOF/VEL in children 6-18 years of age. Despite the decrease in BMI Z score in boys observed at 12 weeks after treatment, no differences were found between baseline and 1-year posttreatment values. Our observations confirm the long-term safety of the SOF/VEL treatment in children 6-18 years of age.

## INTRODUCTION

Direct-acting antivirals (DAAs) caused a breakthrough in the treatment of chronic hepatitis C in children and adolescents.^1^ Sofosbuvir and velpatasvir (SOF/VEL) combination pangenotypic therapy leads to improved clinical outcomes and sustained viral response (SVR) in adults.^2–4^ Available clinical data in children are limited, but they suggest similar efficacy independently of hepatitis C virus (HCV) genotypes, making SOF/VEL considered for the first-line therapy.^5–9^ In addition, this combination is well tolerated with only mild-to-moderately severe adverse events.^5–7,9^ Thus, SOF/VEL has emerged as a highly effective and safe alternative to traditional interferon–based therapies previously used in children and adolescents. SOF/VEL combination offers higher SVR rates, a shorter treatment duration, an oral regimen and a better safety profile. In addition, interferon-based therapies had a significant negative impact on children’s growth, leading to the deterioration of body weight, growth and body mass index (BMI).^10^ This effect was generally reversible; however, long-term influence on the children’s height was reported for over 2 years after treatment with pegylated interferon.^10^ As current published data on the outcomes of SOF/VEL treatment in children and adolescents are limited, there are still gaps in our knowledge.^6^ In particular, the long-term influence of this therapy on children’s growth and its potential effect on their development have not been studied so far. On the other hand, data from adult studies indicate a possible impact of DAAs on dyslipidemia and a significant increase in BMI after HCV eradication.^11–14^ Thus, the aim of this study was to evaluate the influence of treatment of hepatitis C with SOF/VEL on children’s growth parameters in the whole treated group and in subgroups according to the sex and age of the participants.

## METHODS

Fifty children 6-18 years of age were successfully treated for hepatitis C with a 12-week course of SOF/VEL fixed dose adjusted to the body weight in the PANDAA-PED project described in detail recently.^7^ In brief, the PANDAA-PED study (Treatment of chronic hepatitis C in children 6–18 years of age using a pangenotypic direct-acting antiviral sofosbuvir/velpatasvir) is a noncommercial, nonrandomized, open-label study founded by the Medical Research Agency, Warsaw, Poland (grant number 2019/ABM/01/00014). Recruitment for this study lasted since January 2022, and all the children completed their SVR evaluation before December 31, 2022. The primary endpoints of this study included an assessment of the SOF/VEL efficacy and safety in children 6-18 years of age. Among secondary endpoints, there was the analysis of the influence of the treatment on children’s growth. In this part of the study, we analyzed the results of the 1-year posttreatment visits, which were completed by October 31, 2023. Only children who underwent all the scheduled visits were included in this analysis.

In the present part of the study, we compared growth parameters (BMI, height and weight) at 1 year after treatment with baseline (at the start of treatment) and 12-week-posttreatment values. As these parameters may be influenced by other factors, including pubertal growth, Z scores adjusted to sex and age were calculated for BMI, weight and height according to the World Health Organization growth reference data for children 5-19 years of age (https://www.who.int/tools/growth-reference-data-for-5to19-years, data accessed: February 7, 2024). Z score values were compared to establish whether the differences in the growth parameters were appropriate. The following interpretation of BMI Z score cutoffs proposed by the World Health Organization was used: overweight: >+1 SD (equivalent to BMI of 25 kg/m^2^ at 19 years); obesity: >+2 SD (equivalent to BMI of 30 kg/m^2^ at 19 years) and thinness: <−2 SD. Weight Z scores were calculated only for children up to 10 years of age, as weight-for-age reference data are not available beyond the age of 10 years because this indicator does not distinguish between height and body mass in an age period where many children are experiencing the pubertal growth spurt.

### Statistical Analysis

Continuous data were tested for normal distribution using the Kolmogorov-Smirnov test and were presented as means (95% confidence intervals) or medians with interquartile ranges. To compare the parameters between the subsequent visits, repeated measures analysis of variance testing was used. Categorical variables were expressed as numbers (percentages of total), and they were compared using a χ^2^ test. A 2-sided *P* value of <0.05 was considered to indicate significance. Statistical analyses were performed using MedCalc Statistical Software, version 22.018 (MedCalc, Ostend, Belgium).

### Ethical Statement

Written informed consent was collected from all the patients and/or their parents/guardians before their inclusion in the study. The local ethics committee of the Medical University of Warsaw approved this study (approvals number KB/136/2020, September 14, 2020, and KB/30/A2021, April 19, 2021). The investigation was performed in accordance with the ethical standards in the 1964 Declaration of Helsinki and its later amendments.

## RESULTS

### Participants

Forty-nine participants (23 boys and 26 girls) of 50 treated children completed all the visits. One 11-year-old girl with normal BMI at the start of treatment was lost to follow-up after the 12-week posttreatment visit. The mean age of the study participants at 1 year after treatment was 10.9 ± 2.5 years, and all children had undetectable HCV RNA at this point. Characteristics of the study group at 1 year after treatment are presented in Table 1.

**TABLE 1.** Characteristics of the Study Group at 1 Year After the End of Treatment With Sofosbuvir/Velpatasvir

Characteristics	Patients (N=49)
Sex, male/female	23 (47%)/26 (53%)
Age, yr	10.9 ± 2.5
Alanine aminotransferase, IU/mL	18 (15–21)
Aspartate aminotransferase, IU/mL	29 (24–33)
Total bilirubin, µmol/L	9.6 (7.6–12.5)
HCV RNA PCR undetectable	49 (100%)
Anti-HCV positive	48 (98%)

Data are presented as numbers (percentage), mean ± standard deviation, or median (interquartile range), as appropriate.

PCR indicates polymerase chain reaction.

### Growth Parameters in the Whole Study Group

At baseline, 33% of participants were overweight or obese, 4% were thin and 63% were normal. This BMI Z score distribution did not change significantly after the treatment (Table 2).

**TABLE 2.** Distribution of Different BMI Z Scores Among Study Group at Baseline (0), 3 Months After the End of Treatment (1) and 12 Months After the End of Treatment (2)

BMI Z score	0	1	2
<−2 SD (thin)	2 (4)	1 (2)	2 (4)
−2 to 1 SD (normal)	31 (63)	33 (67)	30 (61)
≥1 SD (overweight)	12 (25)	12 (25)	15 (31)
≥2 SD (obese)	4 (8)	3 (6)	2 (4)

Data are presented as numbers (percentage); *P* = 0.94.

**Figure 1.**
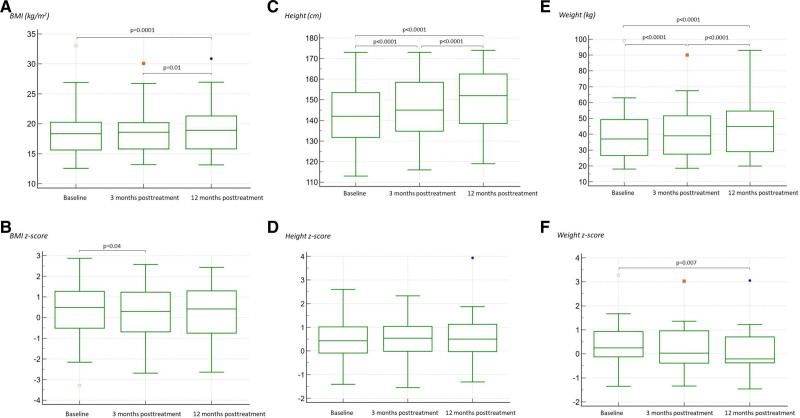
Box-and-whisker plots for growth parameters in 49 pediatric patients before and after treatment with sofosbuvir/velpatasvir: BMI (A), BMI Z score (B), height (C), height Z score (D), weight (E) and weight Z score (F; data available for 16 patients up to 10 years of age). The top and bottom of each box are the 25th and 75th percentiles. The line through the box is the median, and the error bars are the maximum and minimum. *P* values were presented only if they were <0.05.

There was a significant increase in children’s mean height and weight from baseline to both 12 weeks and 1 year after treatment (Table 3; Fig. 1). Height Z scores did not differ during the study periods. For children younger than 10 years, a significant decrease in the mean weight Z score from 0.41 to 0.27 between baseline and 1 year after treatment was observed (*P* = 0.007). The mean BMI at 1 year after treatment was 19.0, and it was significantly higher compared to the baseline and 12-week-posttreatment values (18.6, *P* = 0.01; 18.5, *P* = 0.0001, respectively). BMI Z score decreased from baseline to 12 weeks after treatment (from 0.39 to 0.26, *P* = 0.04), but there was no difference in the value at 1 year after treatment (0.29, *P* = 0.26 compared to baseline, and *P* = 1.0 compared to 12 weeks after treatment).

**TABLE 3.** Growth Parameters in 49 Pediatric Patients Treated With Sofosbuvir/Velpatasvir: At Baseline (0), 3 Months After the End of Treatment (1) and 12 Months After the End of Treatment (2)

Parameter	0	1	2	*P* (0 vs. 1)	*P* (0 vs. 2)	*P* (1 vs. 2)
BMI, kg/m^2^	18.6 (17.4 to 19.7)	18.5 (17.4 to 19.6)	19.0 (17.9 to 20.2)	1.0	0.01	0.0001
BMI Z score	0.39 (0.02 to 0.76)	0.26 (−0.09 to 0.61)	0.29 (−0.04 to 0.64)	0.04	0.26	1.0
Height, cm	143.2 (138.9 to 147.4)	146.4 (142.2 to 150.7)	150.3 (146.0 to 154.5)	<0.0001	<0.0001	<0.0001
Height Z score	0.49 (0.25 to 0.74)	0.53 (0.31 to 0.76)	0.53 (0.26 to 0.80)	0.53	1.0	1.0
Weight, kg	39.5 (35.1 to 43.9)	41.4 (36.8 to 45.4)	44.5 (39.9 to 49.1)	<0.0001	<0.0001	<0.0001
Weight Z score^*^	0.41 (−0.16 to 0.99)	0.27 (−0.29 to 0.84)	0.17 (−0.39 to 0.75)	0.13	0.007	0.22

Data are presented as means (95% confidence intervals). To compare the parameters between the subsequent visits, repeated measures analysis of variance testing was used. Weight Z score calculators are available only for patients below 10 yr of age (16 participants in this study group).

* Weight Z scores were calculated only for children up to 10 years of age, as weight-for-age reference data are not available beyond the age of 10 years.

### Growth Parameters According to Participants’ Age

To analyze the differences in growth parameters according to the patients’ age, participants were divided into 2 groups: younger children (6–11 years of age; n=32) and older children (12–18 years of age; n=17). Growth parameters in these 2 subgroups during subsequent visits were presented in Table 4. In both groups, an increase in the children’s height was observed, without any significant changes in the height Z score, suggesting no influence of the treatment on these parameters and a normal increase in participants’ height. In the younger group, an increase was observed in children’s weight, but the weight Z score decreased at 1 year after treatment compared to baseline, suggesting an insufficient weight gain during this period. However, the BMI at 1 year after treatment was higher compared to the baseline and 12 weeks after treatment, and no significant changes in BMI Z score were reported after treatment. In the older group, the participants’ weight increased significantly at 1 year after treatment, but the increase was nonsignificant between baseline and 12 weeks after treatment. BMI and BMI Z scores did not vary significantly between the studied periods in this group (Table 4).

**TABLE 4.** Growth Parameters According to the Age Group in Pediatric Patients Treated With Sofosbuvir/Velpatasvir: At Baseline (0), 3 Months After the End of Treatment (1) and 12 Months After the End of Treatment (2)

Parameter	0	1	2	*P* (0 vs. 1)	*P* (0 vs. 2)	*P* (1 vs. 2)
Patients 6–11 yr of age (n=32)						
BMI, kg/m^2^	16.8 (15.8 to 17.7)	16.8 (15.8 to 17.7)	17.3 (16.2 to 18.3)	1.0	0.009	0.003
BMI Z score	0.13 (0.33 to 0.59)	0.01 (−0.43 to 0.45)	0.06 (−0.35 to 0.49)	0.32	1.0	0.93
Height, cm	135.0 (131.6 to 138.7)	138.1 (134.3 to 142.0)	142.2 (138.1 to 146.5)	<0.0001	<0.0001	<0.0001
Height Z score	0.56 (0.26 to 0.86)	0.56 (0.27 to 0.85)	0.54 (0.18 to 0.91)	1.0	1.0	1.0
Weight, kg	31.1 (28.1 to 34.1)	32.7 (29.4 to 35.9)	35.8 (32.0 to 39.7)	<0.0001	<0.0001	<0.0001
Weight Z score^*^	0.41 (−0.16 to 0.99)	0.27 (−0.29 to 0.84)	0.17 (−0.39 to 0.75)	0.13	0.007	0.22
Patients 12–18 yr of age (n=17)						
BMI, kg/m^2^	21.9 (19.8 to 24.1)	21.7 (19.8 to 23.7)	22.3 (20.3 to 24.3)	1.0	0.90	0.05
BMI Z score	0.90 (0.31 to 1.49)	0.74 (0.16 to 1.31)	0.72 (0.13 to 1.31)	0.15	0.25	1.0
Height, cm	158.5 (154.6 to 162.5)	162.1 (158.7 to 165.4)	165.2 (162.2 to 168.3)	<0.0001	<0.0001	0.0014
Height Z score	0.37 (−0.07 to 0.82)	0.49 (0.07 to 0.90)	0.50 (0.06 to 0.93)	0.16	0.57	1.0
Weight, kg	55.3 (48.7 to 61.9)	57.0 (51.7 to 62.3)	60.8 (55.4 to 66.1)	0.19	0.001	0.0002

Data are presented as means (95% confidence intervals). To compare the parameters between the subsequent visits, repeated measures analysis of variance testing was used.

* Weight Z scores were calculated only for children up to 10 years of age, as weight-for-age reference data are not available beyond the age of 10 years.

### Growth Parameters According to Gender

Table 5 shows the growth parameters in girls (n=26) and boys (n=23). In both groups, a significant gain in weight and height was observed after treatment. No changes were observed in height Z scores for both girls and boys. Weight Z scores (calculated only for children below 10 years of age) did not change in boys, whereas a decrease was found in girls at 1 year after treatment. BMI did not vary between baseline and both posttreatment visits, but a significant increase was observed between 1-year and 12-week posttreatment assessments in both groups. BMI Z score did not differ significantly among girls, but a decrease was reported in BMI Z score in boys at 12 weeks after treatment compared to the baseline visit.

**TABLE 5.** Growth Parameters According to Sex in Pediatric Patients Treated With Sofosbuvir/Velpatasvir: At Baseline (0), 3 Months After the End of Treatment (1) and 12 Months After the End of Treatment (2)

Parameter	0	1	2	*P* (0 vs. 1)	*P* (0 vs. 2)	*P* (1 vs. 2)
Girls (n=26)						
BMI, kg/m^2^	18.8 (17.0 to 20.6)	18.9 (17.2 to 20.6)	19.3 (17.5 to 21.1)	1.0	0.06	0.009
BMI Z score	0.36 (−0.13 to 0.87)	0.31 (−0.14 to 0.77)	0.28 (−0.17 to 0.75)	1.0	0.83	1.0
Height, cm	144.4 (137.6 to 151.3)	147.3 (140.6 to 154.1)	150.6 (144.2 to 157.1)	<0.0001	<0.0001	<0.0001
Height Z score	0.61 (0.29 to 0.93)	0.66 (0.36 to 0.96)	0.64 (0.30 to 0.98)	0.62	1.0	1.0
Weight, kg	41.2 (33.9 to 48.5)	42.8 (35.9 to 49.6)	45.6 (38.5 to 52.7)	0.01	<0.0001	<0.0001
Weight Z score^*^ (n=9)	0.19 (−0.33 to 0.73)	0.11 (−0.49 to 0.67)	to 0.03 (−0.58 to 0.51)	1.0	0.08	0.04
Boys (n=23)						
BMI, kg/m^2^	18.3 (16.7 to 19.8)	18.1 (16.6 to 19.6)	18.7 (17.1 to 20.2)	0.95	0.31	0.01
BMI Z score	0.43 (−0.15 to 1.02)	0.20 (−0.37 to 0.79)	0.30 (−0.24 to 0.85)	0.02	0.59	0.62
Height, cm	141.7 (136.5 to 146.9)	145.4 (139.9 to 151.0)	149.8 (143.8 to 155.8)	<0.0001	<0.0001	<0.0001
Height Z score	0.36 (−0.02 to 0.75)	0.39 (0.03 to 0.76)	0.40 (−0.04 to 0.85)	1.0	1.0	1.0
Weight, kg	37.6 (32.6 to 42.7)	39.3 (33.9 to 44.7)	43.2 (37.2 to 49.3)	0.0009	<0.0001	<0.0001
Weight Z score^*^ (n=7)	0.70 (−0.65 to 2.05)	0.47 (−0.85 to 1.81)	0.45 (−0.86 to 1.77)	0.19	0.17	1.0

Data are presented as means (95% confidence intervals).

* Weight Z scores were calculated only for children up to 10 years of age, as weight-for-age reference data are not available beyond the age of 10 years.

## DISCUSSION

According to the available data, the SOF/VEL combination is equally effective and safe in children compared to adults.^9,15^ Any potential impact on growth and development could not have been studied in adult patients. Thus, data on the influence of different DAA combinations on children’s growth are only limited, but most of them suggested no negative impact of the therapy on the patients’ growth parameters.^5,16–21^

Our study demonstrated significant weight and height gain after treatment with SOF/VEL irrespective of the patients’ age and sex. As changes in weight and height of pediatric participants may be affected by other factors, including pubertal growth, Z score values adjusted to participants’ age and sex were calculated to make the results more reliable. Height Z scores did not vary significantly in both 12 weeks and 1 year after treatment, which confirms a normal increase in participants’ height. Weight Z scores were available only for 16 children below 10 years of age, and they showed a significant decrease at 1 year after treatment, in girls, which requires further analysis due to the small number of participants in this subgroup. BMI Z score values decreased at 12 weeks after treatment compared to the baseline in the whole group and in boys, but no difference was found between 1-year posttreatment and baseline BMI Z scores in any of the subgroups. This suggests that the possible influence of SOF/VEL on the BMI Z score is transient. In addition, it should be emphasized that the observed statistically significant differences did not seem to be clinically relevant.

Available data from the preregistration SOF/VEL study by Jonas et al did not show any effects of treatment on growth or development in girls or boys 3 to 17 years of age observed up to 24 weeks after treatment.^5,9^ There was, however, a decrease in weight percentile of −2.1 and BMI percentile of −4.8 observed at the end of treatment in children 3 to 6 years of age, but in older age groups, there was a small increase observed in percentiles for weight, height and BMI, respectively.^5^

Four studies analyzed the impact of another SOF-based DAA combination, SOF/ledipasvir on patients’ growth.^16–19^ They included a total of 324 participants 10-18 years of age, and they reported a significant and sufficient gain of weight and growth after SOF/ledipasvir treatment in patients infected with genotypes 1 and 4 HCV. The longest observation period was 1 year after treatment.^16^ Another study analyzed the impact of SOF/daclatasvir combination on weight and linear growth in adolescent patients with chronic HCV infection, and it did not detect any negative effects of the treatment on the analyzed growth parameters.^20^ In addition, a trend toward increased weight gain was observed, probably due to better appetite, which requires further studies. In our SOF/VEL cohort, a significant weight increase was observed (except the insignificant difference in the age group of 12-18 years between 12 weeks after treatment and baseline), but in younger children, a decrease in weight Z score was observed at 1 year after treatment compared to the baseline, which should be further evaluated in other studies.

There is also evidence available from a DORA study on another DAA combination, glecaprevir/pibrentasvir, suggesting that the treatment of adolescents 12 to 17 years of age does not appear to have an impact on their growth or development.^21^ A mean change of BMI and height Z scores from baseline to 12 weeks after treatment in this study was ±0.1.

On the other hand, interesting data come from adult studies on the influence of DAAs on metabolic patterns, indicating a possible impact of DAAs on dyslipidemia and a significant increase in BMI after HCV eradication.^11–14^ Study by Trifan et al^13^ on 280 patients who achieved SVR12 revealed that after viral eradication, a significant increase in triglyceride levels, cholesterol levels and BMI was observed compared to the baseline. An excessive calorie intake might be responsible for the observed phenomenon. This observation requires further evaluation in children and adolescents after successful DAA treatment.

The analysis of available published data suggests that there are still gaps in our knowledge of the long-term impact of DAAs on children’s growth, in participants <12 years of age. To the best of our knowledge, we present data on the longest-term influence of SOF/VEL on children’s growth. However, some limitations of the study should be pointed out. First, the number of analyzed patients was small compared to adult studies, especially when divided into age and sex subgroups, but pediatric studies on larger groups of participants in this field are limited due to the low prevalence of HCV infection in children. Second, the measured growth parameters may be influenced by other factors, including pubertal growth, bone age or parental height, which were not considered in this study. In addition, we did not analyze the potential metabolic or endocrine factors associated with growth parameters; in particular, lipid profiles were unavailable.

In conclusion, the results of the PANDAA-PED study did not show any significant growth deteriorations up to 1 year after successful treatment with SOF/VEL in children 6-18 years of age. Despite the decrease in BMI Z score in boys observed at 12 weeks after treatment, no differences were found between baseline and 1-year posttreatment values. The decrease in weight Z scores in children below 10 years of age requires further analysis, but it does not seem clinically relevant. Our observations confirm another advantage of DAAs compared to the previously used interferon-based therapies, and they prove the long-term safety of the SOF/VEL treatment in children 6 to 18 years of age.
